# 9-(4-Bromo­phen­yl)-9*H*-carbazole

**DOI:** 10.1107/S1600536814003705

**Published:** 2014-02-22

**Authors:** Paul Kautny, Thomas Kader, Berthold Stöger, Johannes Fröhlich

**Affiliations:** a Institute for Applied Synthetic Chemistry, Division Organic Chemistry, Vienna University of Technology, Getreidemarkt 9/163-OC, A-1060 Vienna, Austria; b Institute for Chemical Technologies and Analytics, Division Structural Chemistry, Vienna University of Technology, Getreidemarkt 9/164-SC, A-1060 Vienna, Austria

## Abstract

In the title mol­ecule, C_18_H_12_BrN, the 4-bromo­phenyl ring is inclined to the mean plane of the carbazole moiety (r.m.s. devation = 0.027 Å) by 49.87 (5)°. In the crystal, molecules stack along [001] and are linked by C—H⋯π interactions forming a corrugated two-dimensional network lying parallel to (100).

## Related literature   

For isostructural crystal structures, see: Saha & Samanta (1999 ▶); Chen *et al.* (2005 ▶). For related carbazole-based crystal structures, see: Kim *et al.* (2011 ▶); Liu *et al.* (2010 ▶); Wu *et al.* (2007 ▶); Chen *et al.* (2012 ▶). For a chemically related non-isostructural compound, see: Xie *et al.* (2012 ▶). For applications of aryl­amines as functional materials, see: Shirota & Kageyama (2007 ▶); Tao *et al.* (2011 ▶); Yook & Lee (2012 ▶); Kautny *et al.* (2014 ▶). For isostructurality, see: Kálmán *et al.* (1999 ▶). For merotypism and its application to organic compounds, see: Ferraris *et al.* (2004 ▶); Stöger *et al.* (2012 ▶). For the synthesis of the title compound, see: Xu *et al.* (2007 ▶).
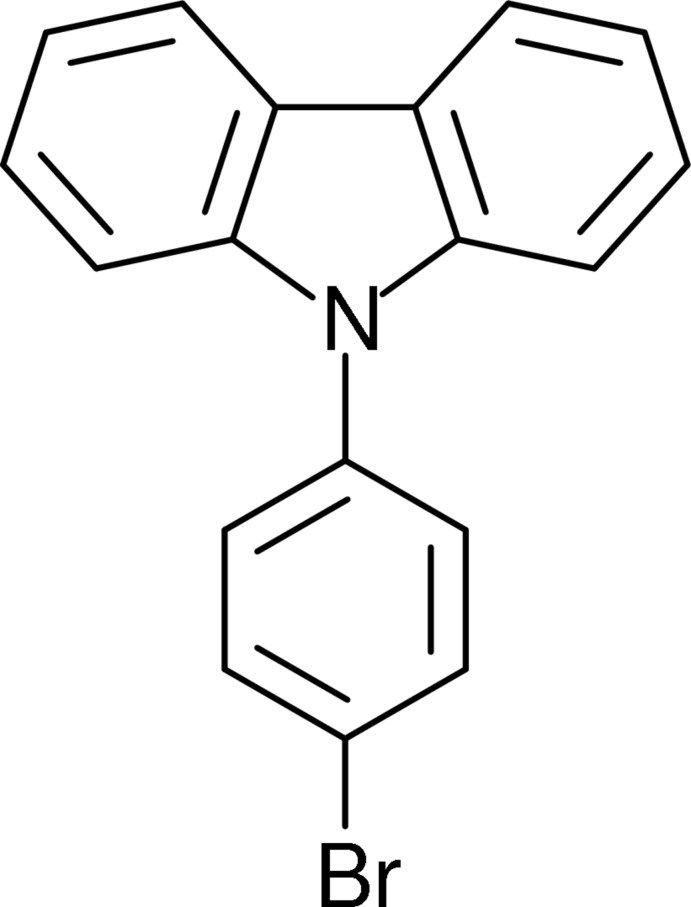


## Experimental   

### 

#### Crystal data  

C_18_H_12_BrN
*M* *_r_* = 322.2
Monoclinic, 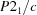

*a* = 8.4137 (3) Å
*b* = 20.1179 (7) Å
*c* = 8.6346 (3) Å
β = 108.5322 (14)°
*V* = 1385.76 (8) Å^3^
*Z* = 4
Mo *K*α radiation
μ = 2.95 mm^−1^
*T* = 100 K
0.75 × 0.55 × 0.42 mm

#### Data collection  

Bruker Kappa APEXII CCD diffractometer
Absorption correction: multi-scan (*SADABS*; Bruker, 2013 ▶) *T*_min_ = 0.16, *T*_max_ = 0.29
53140 measured reflections
5056 independent reflections
4340 reflections with *I* > 3σ(*I*)
*R*_int_ = 0.035

#### Refinement  

*R*[*F* ^2^ > 3σ(*F* ^2^)] = 0.031
*wR*(*F*) = 0.047
*S* = 1.69
5056 reflections
181 parameters
H-atom parameters constrained
Δρ_max_ = 0.47 e Å^−3^
Δρ_min_ = −0.51 e Å^−3^

### 

Data collection: *APEX2* (Bruker, 2013 ▶); cell refinement: *SAINT-Plus* (Bruker, 2013 ▶); data reduction: *SAINT-Plus*; program(s) used to solve structure: *SUPERFLIP* (Palatinus & Chapuis, 2007 ▶); program(s) used to refine structure: *JANA2006* (Petříček *et al.*, 2006 ▶); molecular graphics: *ATOMS* (Dowty, 2006 ▶); software used to prepare material for publication: *publCIF* (Westrip, 2010 ▶).

## Supplementary Material

Crystal structure: contains datablock(s) global, I. DOI: 10.1107/S1600536814003705/kj2237sup1.cif

Structure factors: contains datablock(s) I. DOI: 10.1107/S1600536814003705/kj2237Isup2.hkl

Supporting information file. DOI: 10.1107/S1600536814003705/kj2237Isup3.cml

CCDC reference:

Additional supporting information:  crystallographic information; 3D view; checkCIF report

## Figures and Tables

**Table 1:** Hydrogen-bond geometry (Å, °) *Cg*1, *Cg*3 and *Cg*4 are the centroids of the N1/C7/C12/C13/C18, C7–C12 and C13–C18 rings, respectively.

*D*—H⋯*A*	*D*—H	H⋯*A*	*D*⋯*A*	*D*—H⋯*A*
C3—H1C3⋯*Cg*3^i^	0.96	2.57	3.3237 (14)	135
C5—H1C5⋯*Cg*4^ii^	0.96	2.96	3.7527 (15)	141
C14—H1C14⋯*Cg*1^iii^	0.96	2.79	3.5367 (14)	135

Symmetry codes: (i) 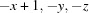; (ii) 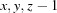; (iii) 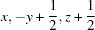.

**Fig. 1.**
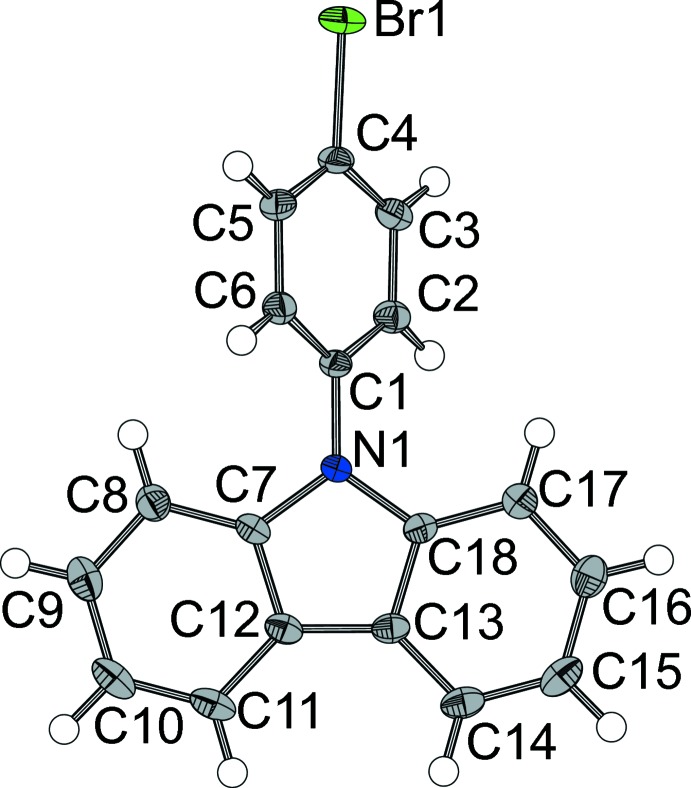
The molecular structure of (I). C, N and Br are represented by grey, blue and green ellipsoids drawn at the 75% probability levels, H atoms by white spheres of arbitrary radius.

**Fig. 2.**
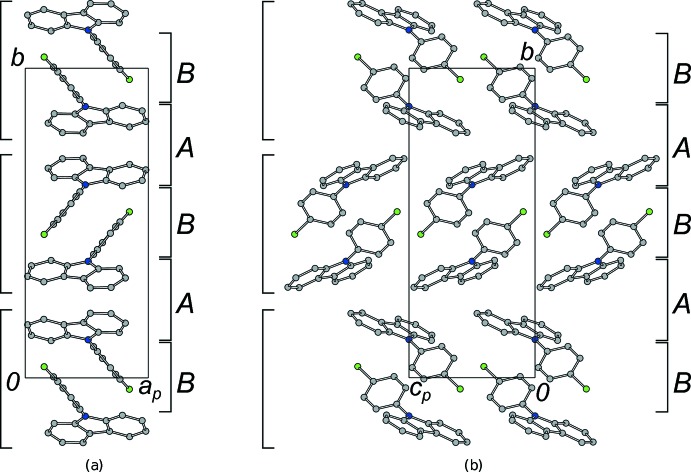
Crystal structure of (I) viewed down (a) [100] and (b) [001]. C, N and Br atoms are represented by spheres of arbitrary radius. H atoms were omitted for clarity. Colour codes as in Fig. 1. The position of the crystallo-chemical layers is indicated to the left, the position of the A and B slabs to the right

**Fig. 3.**
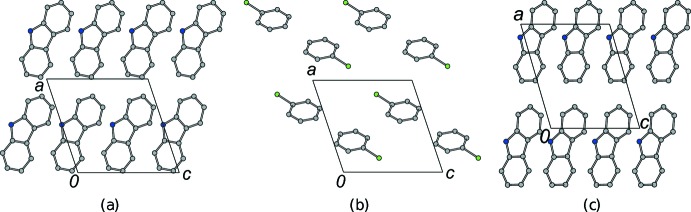
The (a) A and (b) B slabs of (I) composed of 9H-carbazol-9-yl and 4-bromophenyl fragments, respectively projected on (001). (c) The A slabs in 9-(4-(2-(4-(2,1,3-benzothiadiazol-4-ylethynyl)phenyl)vinyl)phenyl)-9H-carbazole (coordinates from Chen, et al., (2012)). Colour codes as in Fig. 1.

**Fig. 4.**
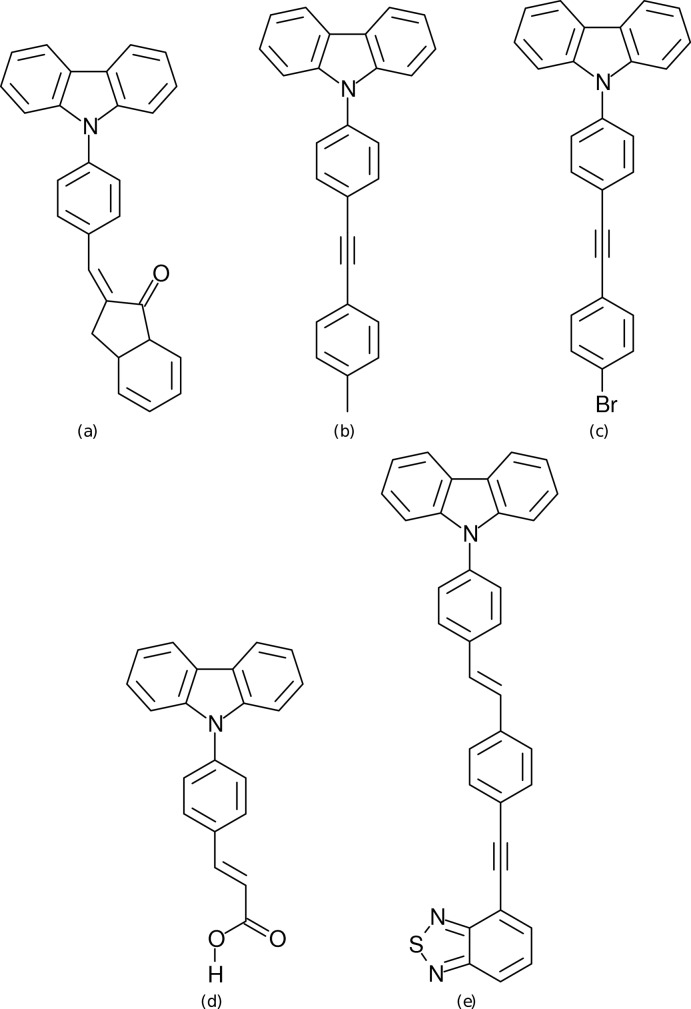
4-(9H-carbazol-9-yl)-phenyl derivatives crystallizing with A slabs isotypic to (I).

**Fig. 5.**
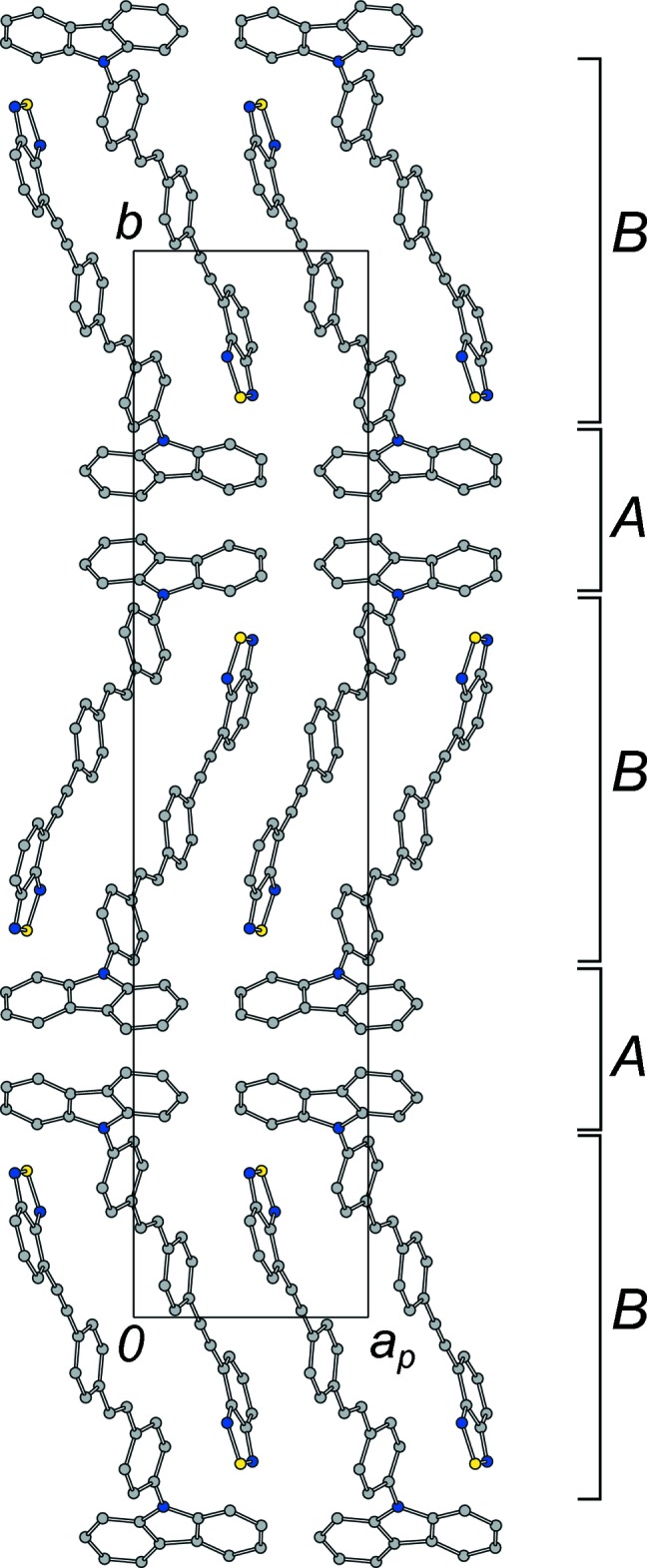
The crystal structure of 9-(4-(2-(4-(2,1,3-benzothiadiazol-4-ylethynyl)phenyl)vinyl)phenyl)-9H-carbazole. S atoms are yellow, other colour codes as in Fig. 1.

## supplementary crystallographic information

### 1. Comment

Arylamines are widely used as electron donors in functional organic materials (Shirota & Kageyama, 2007; Tao *et al.*, 2011; Yook & Lee, 2012). In our recent work we investigated the effect of increasingly planarized triarylamine donors (from *N*,*N*-diphenylbenzenamine to 9-phenyl-9*H*-carbazole and indolo[3,2,1-*jk*]carbazole) on material properties of bipolar compounds (Kautny *et al.*, 2014). Thus, the title compound 9-(4-bromophenyl)-9*H*-carbazole (I) was synthesized as a key intermediate towards materials incorporating 9-phenyl-9*H*-carbazole donor subunits.

(I) crystallizes in the space group *P*2_1_/*c* with one molecule (Fig. 1) in the asymmetric unit. The molecules are arranged in distinct crystallo-chemical layers parallel to (010). The layers contact *via* the carbazoles (Fig. 2 (a,b)). The contacting carbazoles are strongly inclined to each other [angle between least squares (l.s.) plane 59.08°], thus π-π interactions can be ruled out. The phenyl rings inside the layers are related by inversion and therefore coplanar. Nevertheless, the rings do not overlap, excluding π-π interactions (Fig. 2(*b*)).

Two structures that can be considered as isostructural (Kálmán *et al.*, 1999) with (I) have been described, *viz*. the analogues with Br substituted by the pseudo-halogenide CN (Saha and Samanta, 1999) or by a NO_2_ group (Chen *et al.*, 2005). A second polymorph of the CN analogue is structurally unrelated (Xie *et al.*, 2012). In all three isostructural crystals the benzene ring is strongly inclined with respect to the carbazole moiety [angles between l.s. planes of both aromatic systems: (I): 49.87 (5)°; CN: 47.89 (6)°; NO_2_: 53.08 (6)°]. The inclination of the carbazole and phenyl moieties is of particular interest, since it determines the overall degree of conjugation and therefore greatly influences the electro-chemical and photo-physical properties of bipolar materials (Tao *et al.*, 2011).

Several structures with distinctly more bulky substituents on the *para*-position of the phenyl ring have been described which nevertheless feature a virtually identical arrangement of the carbazole rings as observed in (I). Thus, it is useful to ,,slice'' the crystal structure of (I) into two kinds of slabs parallel to (010) which do not correspond to layers in the crystallo-chemical sense as depicted in Fig. 1. The slabs designated as *A* are made up of the carbazole rings of two adjacent crystallo-chemical layers (Fig. 3(*a*)), whereas the *B* slabs are composed of the 4-bromophenyl moieties (Fig. 3(*b*)). The *A* and *B* slabs feature *p*1(*c*)1 (the parentheses mark the direction missing translational symmetry) and *p*1 layer symmetry, respectively. Examples of structures which feature isostructural *A* slabs and structurally unrelated *B* slabs are 2-(4-(9*H*-carbazol-9-yl)benzylidene)indan-1-one (Fig. 4(*a*)) (Kim *et al.*, 2011), 9-(4-((4-methylphenyl)ethynyl)phenyl)-9*H*-carbazole (Fig. 4(*b*)) and the isostructural 4-bromophenyl analogue (Fig. 4(*c*)) (Liu *et al.*, 2010), the mono-toluene solvate of 3-(4-(9*H*-carbazol-9-yl)phenyl)acrylic acid (Fig. 4(*d*)) (Wu *et al.*, 2007) and 9-(4-(2-(4-(2,1,3-benzothiadiazol-4-ylethynyl)phenyl)vinyl)phenyl)-9*H*-carbazole (Fig. 4(*e*)) (Chen, *et al.*, 2012). The crystal structure of the latter is depicted in Fig. 5 and a comparison of the *A* slabs to those in (I) is given in Fig. 3(*c*). Despite being structurally unrelated, the *B* slabs in all these structures feature, like the corresponding slab in (I), *p*1 symmetry. Therefore, these structures possess likewise overall *P*2_1_/*c* space group symmetry. In the crystal chemistry of inorganic compounds, the *A* and *B* slabs are called modules and the structures given above can be considered as members of a merotypic series (Ferraris *et al.*, 2004). Whereas describing crystal structures of organic molecules in terms of modular materials is uncommon, we have recently applied these concepts to the solvatomorphs of a carbazole based organic molecule related to (I) (Stöger *et al.*, 2012).

### 2. Experimental

The synthesis of (I) was performed according to the procedure described by Xu *et al.* (2007). A fused silica ampoule was charged with 9*H*-carbazole (5.35 g, 32.0 mmol, 1.00 eq.), 1,4-dibromobenzene (9.06 g, 38.4 mmol, 1.20 eq.), CuSO_4_·5H_2_O (400 mg, 1.6 mmol, 0.05 eq.) and K_2_CO_3_ (4.42 g, 32.0 mmol, 1.00 eq.) The sealed ampoule was heated to 250 °C for 68 h. After cooling, the tube was carefully opened with a diamond blade, releasing a small amount of gas. The solid residue was partitioned between toluene and water and the aqueous phase was extracted with toluene. The combined organic layers were washed with water, dried over anhydrous Na_2_SO_4_ and concentrated under reduced pressure. Purification was performed by column chromatography (light petroleum:DCM 75:25) yielding 9-(4-bromophenyl)-9*H*-carbazole (4.22 g, 13.1 mmol, 41%) as white solid. Large single crystals of (I) were grown by slow evaporation of a CDCl_3_ solution.

### 3. Refinement

The structure was refined against *F* values using the Jana2006 software package (Petříček *et al.*, 2006). The non-H atoms were located in the electron density map obtained by charge-flipping implemented in SUPERFLIP (Palatinus & Chapuis, 2007) and refined with anisotropic displacement parameters. The H atoms were placed at calculated positions and refined as riding on the parent C atoms.

### Crystal data

**Table:**

C_18_H_12_BrN	*F*(000) = 648
*M**_r_* = 322.2	*D*_x_ = 1.544 Mg m^−^^3^
Monoclinic, *P*2_1_/*c*	Mo *K*α radiation, λ = 0.71073 Å
Hall symbol: -P 2ycb	Cell parameters from 25237 reflections
*a* = 8.4137 (3) Å	θ = 2.5–32.5°
*b* = 20.1179 (7) Å	µ = 2.95 mm^−^^1^
*c* = 8.6346 (3) Å	*T* = 100 K
β = 108.5322 (14)°	Block, clear colourless
*V* = 1385.76 (8) Å^3^	0.75 × 0.55 × 0.42 mm
*Z* = 4	

### Data collection

**Table:**

Bruker Kappa APEXII CCD diffractometer	5056 independent reflections
Radiation source: X-ray tube	4340 reflections with *I* > 3σ(*I*)
Graphite monochromator	*R*_int_ = 0.035
ω and φ scans	θ_max_ = 32.8°, θ_min_ = 2.6°
Absorption correction: multi-scan (*SADABS*; Bruker, 2013)	*h* = −12→12
*T*_min_ = 0.16, *T*_max_ = 0.29	*k* = −30→30
53140 measured reflections	*l* = −13→13

### Refinement

**Table:**

Refinement on *F*	Primary atom site location: iterative
*R*[*F*^2^ > 2σ(*F*^2^)] = 0.031	Hydrogen site location: inferred from neighbouring sites
*wR*(*F*^2^) = 0.047	H-atom parameters constrained
*S* = 1.69	Weighting scheme based on measured s.u.'s *w* = 1/(σ^2^(*F*) + 0.0004*F*^2^)
5056 reflections	(Δ/σ)_max_ = 0.026
181 parameters	Δρ_max_ = 0.47 e Å^−^^3^
0 restraints	Δρ_min_ = −0.51 e Å^−^^3^
48 constraints	

### Fractional atomic coordinates and isotropic or equivalent isotropic displacement parameters (Å^2^)

**Table:**

	*x*	*y*	*z*	*U*_iso_*/*U*_eq_	
Br1	0.84651 (2)	−0.036314 (8)	−0.405902 (18)	0.02427 (6)	
N1	0.51185 (14)	0.12342 (5)	0.00001 (13)	0.0116 (3)	
C1	0.58687 (16)	0.08548 (6)	−0.09658 (15)	0.0111 (3)	
C2	0.69269 (18)	0.03277 (6)	−0.02622 (17)	0.0133 (4)	
C3	0.77018 (17)	−0.00373 (6)	−0.11813 (17)	0.0139 (4)	
C4	0.73532 (17)	0.01171 (7)	−0.28261 (16)	0.0139 (4)	
C5	0.62817 (17)	0.06309 (7)	−0.35594 (16)	0.0148 (4)	
C6	0.55565 (17)	0.10094 (7)	−0.26077 (15)	0.0135 (3)	
C7	0.34167 (16)	0.13791 (6)	−0.03947 (16)	0.0118 (3)	
C8	0.21016 (17)	0.12051 (7)	−0.17824 (16)	0.0147 (4)	
C9	0.04953 (18)	0.13772 (7)	−0.18154 (18)	0.0174 (4)	
C10	0.01993 (18)	0.17175 (7)	−0.05191 (19)	0.0190 (4)	
C11	0.15091 (18)	0.18942 (7)	0.08536 (18)	0.0172 (4)	
C12	0.31464 (17)	0.17212 (6)	0.09265 (16)	0.0127 (4)	
C13	0.47576 (17)	0.17913 (6)	0.21673 (16)	0.0122 (3)	
C14	0.52962 (19)	0.20908 (6)	0.37103 (17)	0.0164 (4)	
C15	0.6979 (2)	0.20772 (7)	0.46009 (17)	0.0186 (4)	
C16	0.81376 (18)	0.17722 (7)	0.39656 (17)	0.0168 (4)	
C17	0.76424 (17)	0.14676 (6)	0.24474 (16)	0.0143 (4)	
C18	0.59415 (16)	0.14789 (6)	0.15611 (15)	0.0116 (3)	
H1c2	0.712034	0.021682	0.086423	0.016*	
H1c3	0.846464	−0.038992	−0.069149	0.0167*	
H1c5	0.604265	0.072462	−0.470097	0.0178*	
H1c6	0.484129	0.13771	−0.308549	0.0162*	
H1c8	0.229898	0.097553	−0.267885	0.0176*	
H1c9	−0.043798	0.125999	−0.275106	0.0208*	
H1c10	−0.092883	0.182992	−0.058358	0.0228*	
H1c11	0.130161	0.212998	0.17367	0.0206*	
H1c14	0.450589	0.230279	0.414296	0.0197*	
H1c15	0.735971	0.227799	0.566404	0.0223*	
H1c16	0.930439	0.177463	0.459918	0.0202*	
H1c17	0.844122	0.125706	0.202336	0.0172*	

### Atomic displacement parameters (Å^2^)

**Table:**

	*U*^11^	*U*^22^	*U*^33^	*U*^12^	*U*^13^	*U*^23^
Br1	0.02113 (9)	0.03450 (10)	0.02083 (9)	0.00656 (6)	0.01183 (6)	−0.00729 (6)
N1	0.0095 (5)	0.0143 (5)	0.0113 (4)	0.0012 (4)	0.0040 (4)	−0.0020 (4)
C1	0.0106 (5)	0.0134 (5)	0.0109 (5)	−0.0005 (4)	0.0055 (4)	−0.0013 (4)
C2	0.0139 (6)	0.0133 (5)	0.0145 (6)	−0.0009 (4)	0.0069 (5)	0.0000 (4)
C3	0.0127 (6)	0.0132 (5)	0.0174 (6)	0.0004 (4)	0.0071 (5)	−0.0009 (4)
C4	0.0121 (5)	0.0173 (6)	0.0148 (5)	−0.0017 (5)	0.0079 (5)	−0.0046 (5)
C5	0.0147 (6)	0.0199 (6)	0.0116 (5)	−0.0005 (5)	0.0066 (5)	−0.0015 (5)
C6	0.0125 (5)	0.0166 (6)	0.0120 (5)	0.0007 (4)	0.0047 (4)	0.0003 (4)
C7	0.0105 (5)	0.0114 (5)	0.0147 (5)	0.0016 (4)	0.0059 (4)	0.0011 (4)
C8	0.0128 (6)	0.0167 (6)	0.0145 (5)	0.0000 (4)	0.0042 (4)	0.0010 (4)
C9	0.0126 (6)	0.0192 (6)	0.0192 (6)	0.0008 (5)	0.0034 (5)	0.0048 (5)
C10	0.0130 (6)	0.0174 (6)	0.0286 (7)	0.0043 (5)	0.0094 (5)	0.0044 (5)
C11	0.0167 (6)	0.0140 (6)	0.0249 (7)	0.0037 (5)	0.0123 (5)	0.0008 (5)
C12	0.0134 (5)	0.0105 (5)	0.0161 (6)	0.0016 (4)	0.0073 (5)	0.0008 (4)
C13	0.0157 (6)	0.0091 (5)	0.0135 (5)	0.0001 (4)	0.0071 (4)	−0.0005 (4)
C14	0.0222 (7)	0.0117 (5)	0.0172 (6)	0.0004 (5)	0.0090 (5)	−0.0034 (4)
C15	0.0260 (7)	0.0148 (6)	0.0140 (6)	−0.0028 (5)	0.0051 (5)	−0.0039 (5)
C16	0.0181 (6)	0.0151 (6)	0.0158 (6)	−0.0031 (5)	0.0033 (5)	−0.0003 (5)
C17	0.0139 (6)	0.0147 (5)	0.0144 (5)	0.0000 (4)	0.0046 (5)	−0.0003 (4)
C18	0.0133 (5)	0.0109 (5)	0.0113 (5)	−0.0007 (4)	0.0051 (4)	−0.0011 (4)

### Geometric parameters (Å, º)

**Table:**

Br1—C4	1.8909 (15)	C9—C10	1.400 (2)
N1—C1	1.4182 (19)	C9—H1c9	0.96
N1—C7	1.3932 (17)	C10—C11	1.3840 (18)
N1—C18	1.3947 (15)	C10—H1c10	0.96
C1—C2	1.3937 (17)	C11—C12	1.403 (2)
C1—C6	1.3925 (18)	C11—H1c11	0.96
C2—C3	1.387 (2)	C12—C13	1.4430 (17)
C2—H1c2	0.96	C13—C14	1.3999 (18)
C3—C4	1.3910 (19)	C13—C18	1.412 (2)
C3—H1c3	0.96	C14—C15	1.380 (2)
C4—C5	1.3853 (18)	C14—H1c14	0.96
C5—C6	1.395 (2)	C15—C16	1.403 (2)
C5—H1c5	0.96	C15—H1c15	0.96
C6—H1c6	0.96	C16—C17	1.3858 (19)
C7—C8	1.3928 (16)	C16—H1c16	0.96
C7—C12	1.411 (2)	C17—C18	1.3922 (18)
C8—C9	1.387 (2)	C17—H1c17	0.96
C8—H1c8	0.96		
			
C1—N1—C7	125.70 (10)	C10—C9—H1c9	119.15
C1—N1—C18	125.54 (11)	C9—C10—C11	120.97 (14)
C7—N1—C18	108.58 (12)	C9—C10—H1c10	119.51
N1—C1—C2	119.68 (12)	C11—C10—H1c10	119.51
N1—C1—C6	120.22 (11)	C10—C11—C12	118.56 (14)
C2—C1—C6	120.10 (13)	C10—C11—H1c11	120.72
C1—C2—C3	120.32 (13)	C12—C11—H1c11	120.72
C1—C2—H1c2	119.84	C7—C12—C11	119.54 (11)
C3—C2—H1c2	119.84	C7—C12—C13	107.05 (12)
C2—C3—C4	118.76 (12)	C11—C12—C13	133.35 (13)
C2—C3—H1c3	120.62	C12—C13—C14	133.82 (14)
C4—C3—H1c3	120.62	C12—C13—C18	106.75 (11)
Br1—C4—C3	118.63 (10)	C14—C13—C18	119.43 (12)
Br1—C4—C5	119.37 (11)	C13—C14—C15	119.10 (15)
C3—C4—C5	121.92 (14)	C13—C14—H1c14	120.45
C4—C5—C6	118.75 (12)	C15—C14—H1c14	120.45
C4—C5—H1c5	120.62	C14—C15—C16	120.54 (13)
C6—C5—H1c5	120.63	C14—C15—H1c15	119.73
C1—C6—C5	120.10 (12)	C16—C15—H1c15	119.73
C1—C6—H1c6	119.95	C15—C16—C17	121.73 (12)
C5—C6—H1c6	119.95	C15—C16—H1c16	119.13
N1—C7—C8	129.17 (13)	C17—C16—H1c16	119.13
N1—C7—C12	108.74 (10)	C16—C17—C18	117.40 (14)
C8—C7—C12	122.00 (13)	C16—C17—H1c17	121.3
C7—C8—C9	117.23 (13)	C18—C17—H1c17	121.3
C7—C8—H1c8	121.39	N1—C18—C13	108.87 (11)
C9—C8—H1c8	121.39	N1—C18—C17	129.30 (14)
C8—C9—C10	121.70 (12)	C13—C18—C17	121.78 (12)
C8—C9—H1c9	119.15		

### Hydrogen-bond geometry (Å, º)

*Cg*1, *Cg*3 and *Cg*4 are the centroids of the N1/C7/C12/C13/C18, C7–C12 and C13–C18 rings, respectively.

**Table:**

*D*—H···*A*	*D*—H	H···*A*	*D*···*A*	*D*—H···*A*
C3—H1C3···*Cg*3^i^	0.96	2.57	3.3237 (14)	135
C5—H1C5···*Cg*4^ii^	0.96	2.96	3.7527 (15)	141
C14—H1C14···*Cg*1^iii^	0.96	2.79	3.5367 (14)	135

Symmetry codes: (i) −*x*+1, −*y*, −*z*; (ii) *x*, *y*, *z*−1; (iii) *x*, −*y*+1/2, *z*+1/2.

## References

[bb1] Bruker (2013). *SAINT-Plus*, *APEX2* and *SADABS* Bruker AXS Inc., Madison, Wisconsin, USA.

[bb2] Chen, S., Chen, N., Yan, Y.-L., Liu, T., Yu, Y., Li, Y., Liu, H., Zhao, Y.-S. & Li, Y. (2012). *Chem. Commun.* **48**, 9011–9013. 10.1039/c2cc32501b 22655296

[bb3] Chen, L.-Q., Yang, C.-L., Meng, X.-G. & Qin, J.-G. (2005). *Acta Cryst.* E**61**, o3073–o3075.

[bb4] Dowty, E. (2006). *ATOMS* Shape Software, Kingsport, Tennessee, USA.

[bb5] Ferraris, G., Makovicky, E. & Merlino, S. (2004). *Crystallography of Modular Materials*, Vol. 15, *IUCr Monographs on Crystallography* Oxford University Press.

[bb6] Kálmán, A., Párkányi, L. & Argay, G. (1999). *Chem. Commun.* pp. 605–606.

[bb7] Kautny, P., Lumpi, D., Wang, Y., Tissot, A., Bintinger, J., Horkel, E., Stöger, B., Hametner, C., Hagemann, H., Ma, D. & Fröhlich, J. (2014). *J. Mater. Chem. C.* Accepted. 10.1039/C3TC32338B.

[bb8] Kim, B.-S., Kim, S.-H., Shinya, M. & Son, Y. A. (2011). *Z. Kristallogr. New Cryst. Struct.* **226**, 177.

[bb9] Liu, R., Zhu, H., Chang, J., Xiao, Q., Li, Y. & Chen, H. (2010). *J. Lumin.* **130**, 1183–1188.

[bb10] Palatinus, L. & Chapuis, G. (2007). *J. Appl. Cryst.* **40**, 786–790.

[bb11] Petříček, V., Dušek, M. & Palatinus, L. (2006). *JANA2006* Institute of Physics, Praha, Czech Republic.

[bb12] Saha, S. & Samanta, A. (1999). *Acta Cryst.* C**55**, 1299–1300.

[bb13] Shirota, Y. & Kageyama, H. (2007). *Chem. Rev.* **107**, 953–1010. 10.1021/cr050143+ 17428022

[bb14] Stöger, B., Kautny, P., Lumpi, D., Zobetz, E. & Fröhlich, J. (2012). *Acta Cryst.* B**68**, 667–676. 10.1107/S0108768112039651 23165603

[bb15] Tao, Y., Yang, C. & Qin, J. (2011). *Chem. Soc. Rev.* **40**, 2943–2970. 10.1039/c0cs00160k 21369622

[bb16] Westrip, S. P. (2010). *J. Appl. Cryst.* **43**, 920–925.

[bb17] Wu, J.-Y., Pan, Y.-L., Zhang, X.-J., Sun, T., Tian, Y.-P., Yang, J.-X. & Chen, Z.-N. (2007). *Inorg. Chim. Acta*, **360**, 2083–2091.

[bb18] Xie, Y.-Z., Jin, J.-Y. & Qu, X.-C. (2012). *Acta Cryst.* E**68**, o1199. 10.1107/S1600536812012457 PMC3344136 22606139

[bb19] Xu, H., Yin, K. & Huang, W. (2007). *Chem. Eur. J.* **13**, 10281–10293. 10.1002/chem.200700678 17918175

[bb20] Yook, K. S. & Lee, J. Y. (2012). *Adv. Mater.* **24**, 3169–3190. 10.1002/adma.201200627 22641461

